# Is microaggression an oxymoron? A mixed methods study on attitudes toward racial microaggressions among United States university students

**DOI:** 10.1371/journal.pone.0243058

**Published:** 2020-12-02

**Authors:** P. Priscilla Lui, Shalanda R. Berkley, Savannah Pham, Lauren Sanders

**Affiliations:** Department of Psychology, Southern Methodist University, Dallas, Texas, United States of America; Brown University, UNITED STATES

## Abstract

To best understand the possible negative health and social consequences associated with racial microaggression, in-depth understanding of how people judge these events is needed. People of Color (POC) and White participants (*N* = 64) were recruited for a mixed-methods study that incorporated quantitative attitude ratings and focus group interviews. Participants read and discussed their attitudes toward five vignettes that reflected microassault, microinsult, and microinvalidation scenarios. Semantic differential ratings showed that participants judged microassaults to be most unacceptable, followed by microinsults and then microinvalidations. Using a grounded theory approach, our qualitative analysis of interview data revealed five thematic categories. First, participants judged receivers’ psychological harm to be a critical consideration for their attitudes toward microaggression scenarios; they discussed factors associated with individual differences in appraisals, prior exposures to discrimination, and sensitivity to race. Second, participants were less consistent in their opinion about the role of the deliverers’ intent on their judgment of microaggressions; many considered microaggression events to be results of deliverers’ cultural ignorance and racial insensitivity. Third, our analysis revealed the central importance of contexts that shaped participants’ attitudes toward microaggression. Fourth, participants also discussed the notion that receivers of microaggression were racist for calling attention to race issues. Finally, POC participants tended to relate to the vignettes and use their lived experiences to contextualize their opinions about racial microaggression. The current results raise concerns regarding the conceptualization and utility of the word “microaggression,” especially within the broader contexts of racism and major discrimination. Other empirical and practical implications are discussed.

## Introduction

Microaggressions generally refer to everyday exchanges that send insulting or denigrating messages to people from marginalized groups [1, 2]. Over the past decade, microaggression received widespread attention in psychological science and the general public. Research on the nature and possible health impact of microaggression-related experiences has focused on everyday differential treatment experienced by individuals of marginalized group status (e.g., ethnic, gender, and sexual minority). Despite the growth in empirical research and popularity in public discourse, questions about the conceptual clarity of “microaggression” remain. There also appears to be mixed reception of this research program by some scholars and the general public [e.g., 3–7]. In the present study, we examined how individuals judge microaggression scenarios in a sample of university students of diverse backgrounds. We focused on racial microaggressions because the microaggression literature emerged from a critical discussion on race and racism in the American society [1, 2, 8] and most research has focused on microaggressions experienced by People of Color (POC) [9].

### Racism, discrimination, and microaggression

Coined by Chester Pierce, microaggression events were defined as “subtle, stunning, often automatic, and nonverbal exchanges which are ‘putdowns’ of Blacks by offenders” [2]. The concept of microaggression was introduced in the context of racism—an ideology that justified differential treatment of POC groups because of their considered inferiority to Whites [10]. Microaggression marks a public health and mental health problem. According to Pierce, *micro*aggression and *macro*aggression were vehicles for perpetuating systemic racism by inducing guilt, shame, and feelings of inferiority in African Americans [11]. Major discrimination/macroaggression comprises racism-related events (e.g., lynching and denial of civil rights) that are discrete and are likely to be experienced infrequently; everyday discrimination/microaggression comprises minor racism-related stressors (e.g., being treated as a racial stereotype) that are insidious and are likely to be experienced on a daily basis [11, 12]. Given the decreasing rates of major discrimination events in the United States over the past decades, racism has increasingly manifested in subtle and ambiguous discriminatory acts [13, 14]. Still, the public health and psychological significance of microaggression experiences is that these subtle behaviors directed at marginalized groups “often are innocuous,” and place “cumulative weight of their never-ending burden” on African Americans (and other POC) [2].

Research has shown robust associations between POC health outcomes and racism, discrimination, and microaggression experiences [9, 15–24]. Although microaggression and major discrimination were manifestations of racism, they have tended to be examined in separate literatures. One reason may be that microaggressions typically are contrasted to major discrimination events on the basis that deliverers of microaggressive acts are unaware of their racial biases and prejudice. For example, Derald Sue defined racial microaggressions as “everyday slights, insults, putdowns, invalidations, and offensive behaviors that people of color experience in daily interactions with generally well-intentioned White Americans who may be unaware that they have engaged in racially demeaning ways toward target groups” [25]. Still, there appears to be a great deal of overlap in the conceptualizations of microaggression and (everyday) major discrimination.

Within a taxonomy of racial microaggression, Sue categorized common examples of racial and cultural prejudice into microassaults, microinsults, and microinvalidations. He noted that microassaults were “similar to what has been called ‘old fashioned’ racism conducted on an individual level” [1]. By contrast, microinsults were characterized by insensitivity and rudeness that unknowingly snub the receivers, whereas microinvalidations were characterized by unintentional behaviors that negate or nullify the experiences of the receivers [1]. This taxonomy presents two challenges to the conceptualizations of microaggression and everyday major discrimination. On the one hand, considering microaggression to be a manifestation of discrimination and systemic racism in everyday lives [26], it is unclear whether microaggression is distinctive from major discrimination in terms of its conceptual meaning and psychological impact on the receivers. On the other hand, should microassaults be sufficiently different from microinsults and microinvalidations [9, 27], it is unknown whether individuals evaluate and respond to these categories of microaggression events differently.

In addition to conceptual clarity, ambiguity of microaggressive acts raised concerns about whether microaggressions could be verified independently [3]; it was argued that individual differences in the responses to microaggression experiences had not considered the possibly confounding roles of personality traits. Similar to the exposure to other adverse events such as bullying [28], it should not be surprising that individuals might react to microaggression—and other racism-related experiences—differently based on their personal characteristics. Nevertheless, within POC groups, racial microaggression experiences were linked to psychological outcomes over and above trait neuroticism [29, 30]. Research also showed that POC’s health outcomes were disproportionately affected by discrimination and microaggression not because of people’s hypersensitivity to racism; rather, POC were more likely to experience differential treatment relative to Whites [31].

Individual differences in emotional and behavioral responses do not invalidate the delivery of microaggresive acts; yet, receivers (and bystanders) may vary in how they interpret the deliverers’ intent [32]. For example, the higher individuals scored on dispositional forgiveness and public regard for their racial group, the less likely they were to judge the same microaggression scenario to be discriminatory [33, 34]. When the intent of a deliverer of differential treatment was uncertain, individuals were more likely to base their judgment of the interpersonal exchange on whether receivers endured any negative consequences [35]. Still, little research has investigated how intent and harm were associated with individuals’ attitudes toward racial microaggressions.

### Individual differences in perceived intent and psychological harm, and overall attitudes toward microaggression

Existing conceptual confusion around microaggression likely has to do with not only perceived intent and harm, but also the word “micro-*aggression*” itself. According to critics, “aggression” implies that deliverers were explicit in their racial prejudice and intention to offend the receivers, yet, most microaggression examples—particularly microinsults and microinvalidations—were characterized as automatic slights and snubs, and/or well-meaning but insensitive compliments [3, 36]. Lilienfeld suggested that these conceptual concerns invited misuse of the word microaggression [3]. For example, the concept of microaggression has been extended to characterize hierarchies in higher education and adoptive individuals’ experiences [37, 38]. Additionally, researchers had questioned, “to what extent is microaggression a matter that lies in the eye of the beholder, and to what extent does intentionality matter in determining the constitutions of microaggression?” [9].

To advance the understanding of how individuals think about microaggression and its associations with discrimination and racism, the present mixed methods study was rooted in a grounded theory framework [39, 40] and designed to explore answers to three questions: (1) How do people conceptualize the word “microaggression” and defining characteristics of racial microaggression events? (2) To what extent do individuals differ in their judgment of microaggression incidents? (3) How do intent, harm, and ambiguity factor into individuals’ attitudes toward racial microaggression? Consistent with previous research [35], we defined intent as a deliverer’s explicit desire to treat the receiver differentially. We defined harm as negative consequences endured by the receiver as a result of an interpersonal interracial exchange with the deliverer.

## Method

### Participants

Participants were undergraduate and graduate students (*N* = 64; 56% self-identified People of Color) at a predominately White, private university located in Southwestern region of the United States. The sample included Asian Americans (*N* = 15, 63% women, *M*_*age*_ = 19.60, *SD*_*age*_ = 1.12, 80% heterosexual, 87% U.S.-born), Blacks/African Americans (*N* = 9, 55% women, *M*_*age*_ = 19.56, *SD*_*age*_ = 1.24, 89% heterosexual, 89% U.S.-born), Hispanics/Latinx individuals (*N* = 8, 63% women, *M*_*age*_ = 24.38, *SD*_*age*_ = 6.32, 100% heterosexual, 63% U.S.-born), and Whites/Euro Americans (*N* = 27, 52% women, *M*_*age*_ = 20.42, *SD*_*age*_ = 1.50, 92% heterosexual, 96% U.S.-born). Five participants reported mixed ethnoracial backgrounds (Asian/Hispanic, Black/White, Hispanic/White, and White/Native American, and White/Asian; 40% women, *M*_*age*_ = 19.80, *SD*_*age*_ = 0.84, 100% heterosexual, 100% U.S.-born). All but one of the mixed-race participants identified as People of Color.

We focused on university students in our data collection for three reasons. First, race talks and racial prejudice often are heightened and more salient in college than in other areas of life [41, 42]. Second, most studies on microaggressions have been conducted with college-aged adults [9]. Third, many college campuses are engaging in conversations about microaggressions and administering policies to reduce the occurrences and negative impact of racial microaggressions [3]. Individuals were eligible to participate in the present study if they were 18 years or older, and self-identified as POC or Whites/Euro Americans. International students were excluded because racial microaggression reflected power dynamics and historical interracial relations among domestic U.S. racial/ethnic groups, and the existing literature also focused primarily on the experiences in domestic racial/ethnic minority groups.

### Procedures for data collection

The present study was advertised as a focus group investigation that would help advance scientific understanding of interracial relations and racial microaggressions. Participants were recruited through student-directed mass emails (student lists were managed by the Registrar's Office) and the Psychology Subject Pool. Participants from the subject pool received research credits, and names of participants outside of the subject pool were entered into a lottery for one of two $50 electronic gift cards. Participants who were in the subject pool were given the opportunity to choose their preferred option of reimbursement.

We conducted 19 semi-structured focus group interview sessions, 10 of which were with POC participants, and 9 of which were with White participants. A semi-structured interview script was developed and used across interviews (see S1 Text for interview guidelines). This interview format helped reduce the likelihood that moderators’ own personal beliefs affected the types of questions they might ask and allowed flexibility in facilitating discussions among research participants. Race-related dialogues can be difficult, and people often are concerned about how others may evaluate their racial attitudes [42, 43]. For example, individuals may be hesitant in participating in race-related dialogues because they do not want to be seen as racist or insensitive to others. Racially and ethnically matched moderators can reduce cultural mistrust and facilitate participant engagement [44, 45]; therefore, our focus group sessions with White participants were moderated by a Euro American researcher whereas the sessions with POC participants were moderated by an African American researcher. Each focus group interview session included a maximum of 6 participants (range = 2 to 6 because of rescheduling or no-shows) and lasted 90 to 120 minutes. The size of the focus groups was intended to ensure a safe space for rich discussions among participants. Although there were no standard guidelines for the number of participants in qualitative research, we planned to include a minimum of 50 participants from diverse racial backgrounds, and concluded data collection when there were no new emerging themes from focus group discussions.

**Fig 1:**
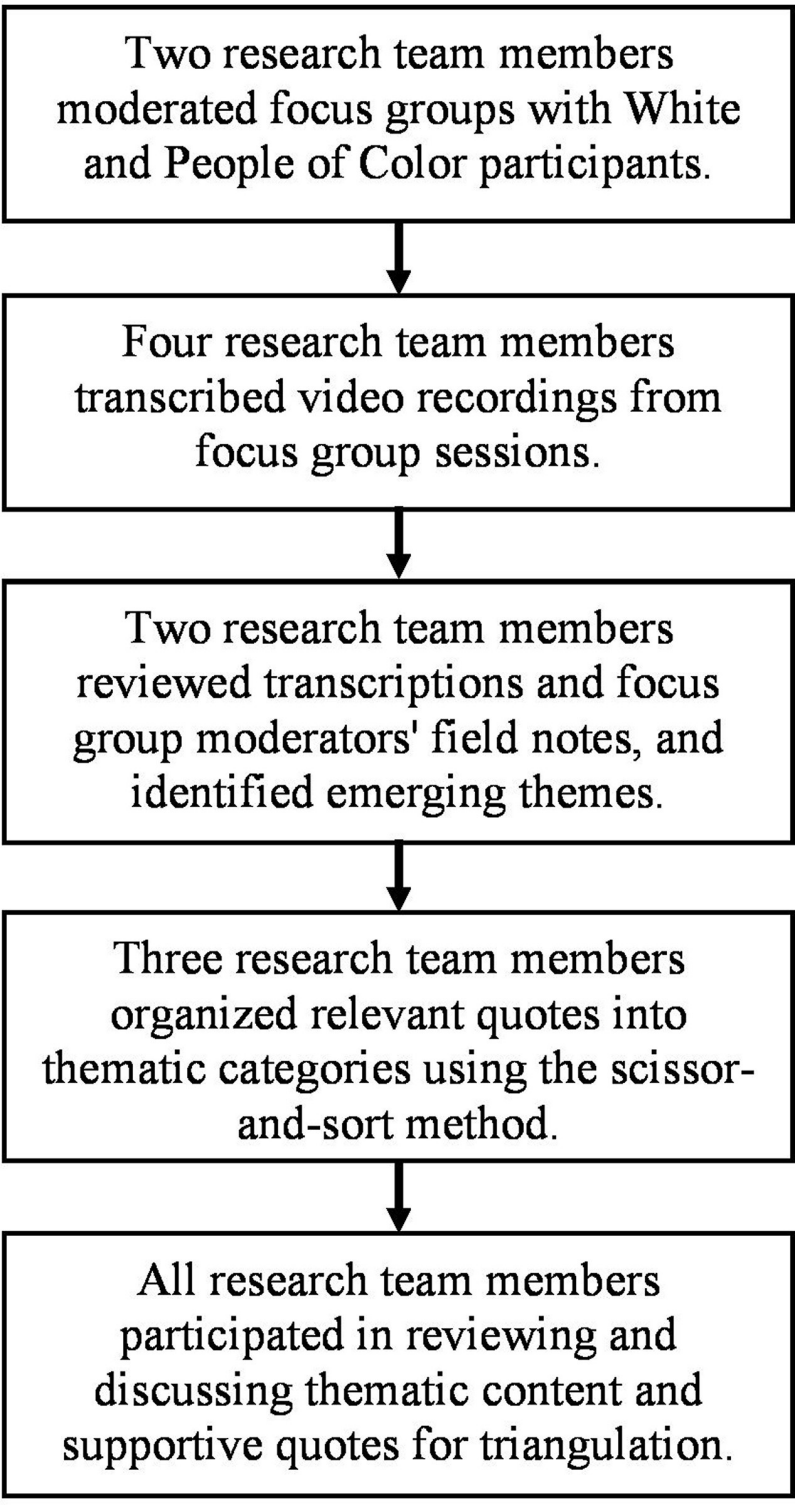
Study procedures that involved focus group interviews, transcription, coding, and thematic content analyses.

The Southern Methodist University Institutional Review Board approved this study. Written consent was obtained from each participant prior to any data collection. Each participant independently read five brief vignettes describing interpersonal interracial exchanges. Participants also provided semantic differential ratings regarding their own opinions about the actors and nature of the interactions in these vignettes (see S2 Text). Upon completing these individual tasks, participants engaged in a moderated focus group discussion. Each participant stated their first name and self-identified racial/ethnic background. They were asked to indicate whether they have had experiences with interpersonal interracial interactions. They were then given the definition of racial microaggression by Sue and colleagues [1], and participated in semi-structured group discussions that were moderated by one researcher. All focus group sessions were video recorded (see Fig 1 for study procedures). Moderators of the focus group interviews followed standard practices in ensuring that participants felt comfortable, encouraging elaboration and discussions, and probing for rich information throughout interviews [46, 47].

### Study materials

Participants read five vignettes that described brief interpersonal exchanges. These vignettes were brief synopses of racial microaggression examples listed in the article by Sue and colleagues [1]. Examples reflected everyday major discrimination/microassault (Scenario 1), as well as microinsult (Scenarios 2 and 3) and microinvalidation (Scenarios 4 and 5). Based on a multidimensional framework of racism, microaggression-related stressors have been compared to major discrimination-related stressors [12]; hence, we presented to participants the written vignettes by order of increasing levels of ambiguity. As the focus group interviews went on, this order likely could facilitate deepened conversations around individuals’ judgments about racial microaggression and discrimination. Major discrimination/microassault situations were defined as conscious and explicit racial derogations; thus, relative to both microinsults and microinvalidations, the major discrimination/microassault vignette was designed to reflect the deliverer’s explicit intent to harm as well as any negative consequences endured by the receivers. For microinsults and microinvalidations, the racial prejudice and intent of the deliverer often are unclear to the receiver and other bystanders; thus, our vignettes reflected the ambiguity of the deliverer’s beliefs. To explore the roles of perceived harm on participants’ judgment, we selected microinsult and microinvalidation vignettes to reflect cases where the receiver appeared bothered/offended (Vignettes 3 and 4), not bothered/offended by the exchange (Vignette 2), and where the receiver’s experience was clear to bystanders (Vignette 5). The vignettes were as follows.

1. A Mexican American heterosexual couple walked into a restaurant. The restaurant was relatively empty. Upon asking for a table, the waiter said to this couple, “We don’t serve people like you. We serve Americans.” The couple was offended and left to visit a different restaurant.
2. At a police precinct, a Black American man walked up to the police officer at the front desk. The White American police officer looked up, and said, “Are you here to turn yourself in for a crime?” The Black American, an accomplished attorney responded, “No, Officer Smith. I am attorney Brown. I am here to represent my client who got arrested this afternoon.” Officer Smith said, “Good for you! Did you become a lawyer after you have been arrested yourself?” Attorney Brown smiled and said, “Oh no! I never had been arrested or even suspected for a crime. I had always wanted to help promote justice that’s all.”
3. During rush hour in New York City, a White American woman and an Asian American woman, both dressed professionally, were waiting at the same spot waiting for a taxi. A White taxi driver saw them both and stopped to pick up the White woman. The Asian American woman thought to herself, “That’s the third time a taxi driver refused to pick me up. This is not cool!”
4. A racially diverse group of college students was discussing whether race had been a salient factor that influenced the most recent election in a Political Science class. A Black student said, “I think race played, and has always played, a critical factor in how people choose their candidates.” A White student said, “I disagree. Race is never a factor. When I think of race, I only think of the human race. I think only the candidates’ qualifications matter.” The Black student responded, “I don’t think you understand my racial experience.”
5. Two friends were talking about a recent promotion at their workplace. Julie, an Asian American woman who had been with the company said, “Only White people get promoted to leadership roles in this company. I don’t think that’s fair.” Julie’s friend, Tom, a White American man said, “I think everyone can succeed if they work hard enough. I think our company should only promote the most qualified people for the job.”

Participants independently rated their reactions to the vignettes on several semantic differential scales. On a 7-point scale, participants rated the degree to which they (a) considered the interaction in each vignette to represent an example of racial microaggression, (b) believed the deliverer in each vignette to have differentially treated, caused harm to, was intentionally insulting to, aware of their behaviors toward, and intentionally hurtful, and racist toward the receiver(s), (c) judged the receiver(s) to be physically or psychological harmed, and (d) thought each interaction to be undesirable and stressful. We computed average scores to indicate participants’ judgment about the deliverer’s intent and overall impression of each vignette.

### Transcription, data coding, semantic content analysis, and validity of qualitative results

Consistent with best practices in qualitative research, moderators kept field notes from all interview sessions to facilitate content analysis [46]. We also followed common procedures to transcribe verbatim all relevant interview responses from participants in each of the focus groups [46, 47]. Information about gestures (e.g., laughter, nod) and other nonverbal cues (e.g., tone of speech) was reflected in the transcriptions as well. Data analysis followed a grounded theory approach and an inductive thematic saturation process [39, 40, 48]. As shown in Fig 1, we conducted coding and content analysis in an iterative fashion. First, the first two authors independently reviewed the transcripts to become familiarized with the data. Second, the first two authors began generating initial themes that might emerge from the data. Third, the coders independently induced potential semantic themes and used the scissor-and-sort technique to organize segments of participants’ responses to assist our interpretation. Fourth, the first three authors reviewed transcripts along with the moderators’ field notes to revise these semantic categories. Fifth, the coding team discussed in research meetings and organized the content themes into broader categories, and identified texts that supported lower-order subthemes.

To ensure the credibility of our coding procedures, the first three authors met regularly to review all selected transcript units and examine the underlying thematic meaning. Other research team members who were not involved in data collection and coding reviewed our themes and supportive texts to triangulate the coding team’s interpretation. These procedures were consistent with the best practices for enhancing the trustworthiness of qualitative analyses [49]. To the extent that qualitative data analysis is a process of meaning making, we determined the adequacy and consistency of our data in terms of the depth of discussions among participants, and in terms of the contextualized information they provided. We did not quantify the number of times that (sub)themes were discussed as to deduce commonality of beliefs and experiences across participants.

### Researcher backgrounds and reflexivity

Our research team consisted of a doctoral-level principal investigator, a doctoral student, and six undergraduate research assistants of diverse racial backgrounds. The principal investigator (first author) is an Asian American psychologist. She has research expertise on discrimination, acculturation, and ethnic disparities in alcohol use and other addictive behaviors. The first author was responsible for the conceptualization of research questions and design of the study protocol, seeking IRB approval, management of the research team, data coding, and writing of the manuscript. At the time of the study, the second author is an African American undergraduate research assistant majoring in psychology and biology. She has research experiences with diversity issues in health outcomes and ethnic health disparities. The second author moderated the focus groups with POC participants, and contributed to the protocol design, transcription, coding, and writing of this research report. The third author is an Asian American doctoral student in clinical psychology. She is interested in acculturation, intergenerational conflict, and psychological adjustment of underserved minority populations. The third author contributed to transcription, coding, and manuscript writing. At the time of the study, the fourth author is a Euro American undergraduate research assistant majoring in psychology. She has research experiences with ethnic diversity issues in mental health. The fourth author moderated focus group sessions with White participants and contributed to the initial preparation for coding and manuscript writing. Other four research team members are of Asian, Latinx, or North African backgrounds with interests in ethnic minority mental health. They transcribed focus group video-recordings, participated regularly in discussions, and contributed to interpretation of coding themes.

Prior to this study, researchers discussed how our experiences and positionality might affect our involvement in the study process. Except the first author, research team members did not have a comprehensive understanding of the racial microaggression research literature. The first three authors reported previous personal experiences with microaggressions; comparatively, the last author was not as aware of the occurrences of microaggressions prior to college. Based on our diverse backgrounds and racialized lived experiences, we expected that participants’ attitudes and evaluations of the vignettes would reflect their personal life experiences. Across both qualitative and quantitative results, we expected to observe systematic differences in the evaluations of vignettes across POC and White participants. Particularly, we thought that White participants might indicate more colorblind attitudes than POC participants whereas POC participants might be more likely to sympathize with the receivers in the vignettes than White participants. Finally, consistent with the conceptualizations around microaggressions, we expected that there would be more differences in opinions across participants about the vignettes that illustrated ambiguous interpersonal exchanges. Whereas the research team believed that participants would discuss issues pertaining to intent and harm, we did not assume any specific thematic content would emerge from our qualitative inquiry. During coding, we engaged in discussions to resolve any discrepancies in the interpretations of quotes from participants and examined the extent to which our understanding reflected researchers’ positionality and backgrounds.

## Results

### Definitions and conceptualizations of discrimination and microaggression

Prior to providing participants with a definition of racial microaggression, individuals were asked to share their understanding of overt, major racial discrimination. Participants considered major discrimination to be ill-intended race-based differential treatment that resulted in negative consequences. Participants believed that major discrimination could occur in interpersonal exchanges as well as through unfair policies and structural biases. For example:

I define [overt discrimination] as intentionally and willfully characterizing or ascribing certain qualities on something that’s necessarily because of their race, and as a consequence treating them differently. Sometimes it also includes creating law or putting laws, procedures, or policies in place which would intentionally harm certain segments of the population solely due [to] their race.–Asian man (22)

Someone actually has to be treated worse, not just different, in a malicious or bad way for it to actually be discrimination.–White man (20)

Except for eight individuals of diverse backgrounds, participants had heard of the term “microaggression” prior to this study. Participants tended to compare microaggressions to overt, major discrimination and describe racial microaggression events as subtler, less severe manifestations of racial stereotypes and prejudices. For example,

[Microaggression] is not like overt racism. Usually it’s because of the culture you’re… its ideas or misperceptions, and it can be well-meaning or accidental racism.–African American man (18)

There are small things that aren’t purposefully racist or discriminatory that if you do it to another race, they’ll find it just not politically correct.–Asian woman (19)

Among participants who were new to the term “racial microaggression,” they nevertheless noted several defining elements of the concept. For example, participants said that,

Typically, a microaggression—rather than a racist encounter—is not motivated ill will; it’s just a lack of understanding [and] knowledge… It might be something that is not taboo, but something that [a] person doesn’t want to hear or be asked.–Hispanic man (20)

Unconscious biases that show up in small interactions. Maybe they are mostly minor incidents but [they] build up over time.–Asian woman (20)

I wouldn’t necessarily say [microaggression] is intended to hurt anyone. Obviously prejudice and discrimination are intended; you know that you are being exclusive and harmful in your words or actions. Microaggression could totally be unintentional; you might just be making an assumption or comment based on what you believe about them—like a stereotype or something.–White woman (19)

Participants converged on common elements that defined microaggressions: everyday experiences with unfair treatment, and actions that were considered rude, insensitive, or insulting to the receivers regardless of the deliverers’ intentions. Both POC and White participants stated that microaggression incidents were small, subtle, and minor aggressive behaviors directed at individuals or groups. Participants believed that these microaggressions typically occurred because of group stereotypes, racial biases, and/or a lack of cultural knowledge.

### Attitudes toward microaggression: Semantic differential ratings

Data on semantic differential ratings were collected to offer complementary information on participants’ judgment of each racial microaggression vignette. As shown in Table 1, relative to other vignettes, participants tended to report more negative attitudes toward Vignette 1 (*M* = 6.74, *SD* = 0.66) and Vignette 2 (*M* = 6.53, *SD* = 0.88). Results showed slightly negative judgment about Vignette 4 (*M* = 3.45, *SD =* 1.48) and neutral evaluation of Vignette 5 (*M* = 3.05, *SD =* 1.44). Participants also thought that there was the highest level of ill-intent by the deliverer in Vignette 1 (*M* = 6.41, *SD =* 1.05*)*, followed by Vignette 2 (*M* = 5.26, *SD =* 1.64) and Vignette 3 (*M* = 4.00, *SD =* 1.39). Participants rated low levels of malicious intent by the deliverers in Scenario 4 (*M* = 2.48, *SD =* 1.31) and Vignette 5 (*M* = 2.33, *SD =* 1.31). Finally, participants indicated that receivers in Vignette 1 (*M* = 5.70, *SD = 0*.*93*), Vignette 2 (*M* = 4.43, *SD =* 1.47), and Vignette 3 (*M* = 4.68, *SD =* 1.34) experienced psychological harm, whereas the receivers in Vignette 4 (*M* = 3.54, *SD =* 1.57) and Vignette 5 (*M* = 3.11, *SD =* 1.39) were not harmed. These ratings were consistent with the definitions for major discrimination/microassaults, microinsults, and microinvalidations. Still, two-way mixed effects intraclass correlation (ICC = .43) indicated that individuals were not in agreement about whether the vignettes were examples of racial microaggression.

**Table 1:** Summary of semantic differential ratings on overall attitudes toward microaggression, and judgment of intent and harm.

	*M* (*SD*)									
	Vignette 1		Vignette 2		Vignette 3		Vignette 4		Vignette 5	
	POC	White	POC	White	POC	White	POC	White	POC	White
**Exemplify Microaggression**	4.44 (2.45)	5.41 (2.15)	6.24 (1.36)	6.48 (1.34)	4.84 (1.69)	5.33 (1.73)	3.57 (2.14)	3.81 (2.13)	3.78 (2.27)	3.00 (2.18)
**Deliverer’s Intent**	6.30 (1.04)	6.38 (0.61)	5.58 (1.03)	5.69 (1.33)	4.40 (1.14)	4.51 (1.62)	2.60 (1.03)	2.65 (1.47)	2.26 (1.20)	2.40 (1.27)
Differential Treatment	6.76 (1.01)	6.74 (1.16)	6.68 (0.75)	6.67 (1.21)	5.41 (1.57)	6.00 (1.65)	.78 (1.62)	2.67 (1.78)	2.19 (1.68)	2.37 (1.98)
Caused harm	5.16 (1.77)	5.41 (1.91)	4.70 (1.81)	5.19 (1.71)	4.19 (1.91)	4.07 (2.13)	2.30 (1.70)	3.07 (2.30)	2.05 (1.43)	2.15 (1.85)
Intentionally insulting	6.35 (1.55)	6.63 (0.69)	5.24 (1.80)	5.22 (1.83)	3.70 (1.63)	3.81 (1.78)	2.19 (1.58)	2.22 (1.45)	1.86 (1.42)	1.96 (1.51)
Aware of Action	6.35 (1.57)	6.52 (1.05)	5.14 (1.89)	5.22 (1.97)	4.16 (1.72)	4.15 (1.85)	2.86 (2.12)	2.74 (1.97)	2.67 (2.12)	3.19 (2.29)
Intentionally Hurtful	6.43 (1.48)	6.19 (1.57)	5.43 (1.57)	5.19 (1.81)	4.24 (1.69)	3.89 (1.40)	2.43 (1.41)	2.41 (1.47)	2.19 (1.37)	2.19 (1.52)
Racist	6.73 (1.02)	6.81 (0.96)	6.30 (1.13)	6.59 (1.22)	4.68 (1.45)	5.15 (1.89)	3.05 (1.79)	2.78 (1.97)	2.54 (1.79)	2.56 (1.72)
**Receiver’s Harm**	5.47 (1.13)	5.63 (1.39)	3.97 (1.69)	4.56 (1.76)	4.00 (1.75)	4.11 (1.95)	3.24 (1.59)	2.85 (1.81)	2.84 (1.89)	2.44 (1.85)
**Miscellaneous**										
Undesirable	6.54 (1.24)	6.96 (0.19)	6.43 (0.96)	6.56 (1.05)	6.00 (1.11)	5.89 (1.25)	4.76 (1.69)	4.59 (1.76)	4.24 (1.82)	4.44 (1.67)
Stressful to Receiver	6.27 (1.22)	6.30 (1.10)	4.54 (1.97)	5.07 (1.80)	5.84 (1.38)	5.89 (1.37)	5.11 (1.53)	4.81 (2.04)	4.84 (1.76)	4.22 (1.65)

*N* = 64. Possible range of all ratings = 1.00 to 7.00. Based on focus group discussions, participants approached the semantic differential rating item for Vignette 1 differently. Whereas participants considered the vignette to be an example of major discrimination and not microaggression per se, they varied in their ratings.

### Attitudes toward microaggression: Responses from focus group discussions

Based on the moderators’ impression, participants showed high levels of engagement during focus group interviews and discussions. All discussions were respectful even when participants disagreed with one another. Participants offered their beliefs, elaborated on their answers, and reacted to other participants’ responses by building on similar thoughts or challenging opposing opinions.

Once they were informed of Sue’s definition of racial microaggression [1], participants indicated the degree to which they believed that there was racist differential treatment involved in each vignette. Participants also shared their perception of each actor that was depicted in the vignettes. Whereas participants stated that each of the five vignettes was brief, they also acknowledged that the lack of details did in fact realistically reflect the ambiguous nature of microaggressions. One 19-year-old African American man said,

“The thing we have to realize is that these conversations happen verbatim; this happens in real life. These are actual conversations that I’m sure I’ve had. You can make assumptions and decide if it is aggressive or not via just these four sentences because it is so real.”

To address each of the three research questions, we followed a grounded theory approach to allow meaning to emerge from qualitative data. Five broad thematic categories emerged from the qualitative data and the first two themes contained subthemes. First, participants believed that the receivers’ perceived harm was a critical factor in their perceptions of microaggressions. Second, participants believed that the deliverers’ perceived intent also mattered in their perceptions of microaggressions. Third, participants believed that the contexts surrounding each of the microaggression vignettes were important in shaping their beliefs and perceptions. Fourth, participants considered receivers to be racist for bringing attention to race issues. Fifth and finally, POC participants tended to use personal experiences to inform their perceptions of microaggressions.

### 1. The meaning of microaggression incidents is determined by receivers’ perceived harm

The most commonly discussed theme across focus group sessions concerned the importance of how microaggression incidents are experienced by receivers. One participant said,

The receiver defines what a microaggression is—whether or not it’s present—and the deliverer’s intention. It can be intended as hurtful but it is whether or not the person interpret[ing] it as a microaggression.–African American man (19)

Participants tended to believe that regardless of how microaggressions occurred, an undesirable interracial exchange existed as long as the receivers experienced psychological harm. Participants considered the receivers’ experiences to be more important than the deliverers’ intent—even if the deliverers or bystanders considered the exchanges to be well-meaning compliments or innocent acts. By contrast, relatively fewer POC and White participants considered perceived harm to be an unimportant consideration. As an illustration, one participant stated that,

The only thing I was thinking is that it was rude or harmful because she [Asian receiver in Vignette 5] might have been saying that with herself like ‘I haven’t gotten the promotion and I work hard,’ so him [White deliverer] being like ‘people who are most qualified for the job will get the promotion’ was harmful to her and putting her down based on qualification. I didn’t get the feeling from this scenario and the information that it was a racial microaggression.–White woman (age unknown)

Participants believed that harm or adverse consequences could manifest in various forms and might affect not only the individual receiver, but also the POC community and society at large.

It’s probably just frustrating than anything [but] not ‘harmful’.–Asian woman (20)

Microaggression isn’t intentionally hurtful but does undermine the experience of the other person.–White woman (21)

Consequently, the harm resulting from those [microaggression] actions still exist even if the person who’s receiving said actions and words doesn’t consider the actions harmful… microaggressions impact an entire ethnicity negatively, so it can go way beyond the receiver even if they are not personally like, ‘oh this hurt me.’–mixed heritage Asian and White woman (20)

When thinking about harm for the receiver, I was thinking more about harm for society as a whole. He [receiver in Vignette 2] didn’t get harmed in that scenario, but the fact of the matter is that mindset as a whole shapes the society that he has to live in… It’s a harmful attitude for society to have because it makes people less aware of something you should be actively aware of.–African American man (18)

Related to the thematic content about receivers’ experiences with microaggressions, our data analysis revealed three subthemes. Specifically, these subthemes concerned individual differences in how receivers react to microaggression-related experiences. In general, participants believed that the reasons for people’s different reactions to the concept of microaggression might have to do with variability in perceiving microaggressions to be stressful, sensitivity to racism and discrimination-related experiences, and the effects of prior exposure to discrimination.

#### 1a. Individual differences in appraisals present challenges in discussing microaggression

Relatively more Whites than POC participants raised the issue of individual differences in responses to racial microaggressions. Participants were unconvinced that in the microaggression vignettes, receivers “left the situation with harm” (19-year-old African American man). Participants believed that individuals differed in the way in which they might perceive, react to, and respond to microaggressions, even when confronted with the same situation. For example,

It really depends on the individual person, their experiences, and how well they are able to brush off these situations.–Asian man (22)

[Microaggression] is one of those things where you definitely think about for the rest of your day… How much they do harm is sort of dependent on how much you let them.–White man (age unknown)

Seeing that [the Black receiver in Vignette 2] smiles and the response that he’s giving, there’s no anger that he reacts with… that could still be putting him down psychologically; he can just cope with [it.]–Asian woman (21)

In addressing why “microaggression” has galvanized a great deal of debate and discussions in the academic and general audiences, participants thought that individual differences in their appraisals and reactions might have made it difficult for people to agree on the negative impact of these interracial exchanges.

#### 1b. People vary in their sensitivity and reactivity to interracial exchanges

Considering adverse consequences of microaggression, participants disagreed on whether receivers of microaggression are overly sensitive or reactive to these incidents because of their appraisals. For example, one participant stated that the receivers in Vignette 4 and Vignette 5 were oversensitive to race:

The Black student [in Vignette 4] took it a little more personal from what the White student had to say. I think the White student was trying to be sensitive about what he had to say, too. [The receiver’s in Vignette 5] emotions get the best of her cognition. She doesn’t think through what exactly she’s saying.–White man (18)

In response to Vignette 3, several participated discussed among themselves:

I think she was being way oversensitive… I don’t know [the taxi situation] totally [had to do with] race.–Asian woman (19)

I don’t think she was overly sensitive to race. I thought her reaction of ‘that’s the third time the taxi drivers refused to pick me up right now, this is not cool.’ I don’t think she was being overly sensitive.–Asian man (22)

In New York City, you have to go and fight for your taxi. If you don’t go actively to the taxi, you shouldn’t wait for them… she shouldn’t blame it upon the first thing she can think of that separates her [race] from other people.–another Asian man (22)

These discussions indicated that POC participants did not uniformly consider receivers’ responses to be valid or justifiable. Among participants who believed the receivers to be oversensitive to race and racism, they tended to judge the vignettes by speculating the receivers’ personality or situational contexts. Furthermore, a small number of POC and White participants expressed doubt about the legitimacy of the microaggression concept.

The cab drivers are just trying to make money… you can’t assume that it’s discrimination or anything like that. They are going to make their money; they are not worried about what you look like in my opinion.–White man (age unknown)

I’ve definitely seen People of Color abuse the word microaggression or claim to be mistreated for their color. People that are blatantly lazy or useless on the job will say, ‘I’m getting fired because I’m Hispanic or because I’m Black.’ No, you can’t even wait tables or you don’t know how to wipe tables down, that’s why you got fired.–Hispanic man (20)

#### 1c. Prior exposure to discrimination might influence the effects of subsequent microaggressions

Participants believed that prior experiences with microaggression or discrimination likely affected how people perceive future microaggression incidents. We observed different opinions about the effects of repeated microaggression experiences, however. Whereas some participants believed that repeated exposures might accumulate the negative effects and lead to worse emotional responses over time, others believed that repeated exposures would desensitize people from reacting negatively. In general, relatively more participants considered prior experiences with discrimination to enhance the negative psychological effects of subsequent exposure. These beliefs were consistent with the definition of microaggressions in the empirical literature.

If it was on a continual basis like every single day, then it would definitely take a toll on her mental state.–African American man (19)

If people keep telling you that [negative stereotypes], you start to internalize that and then it can lead to a lot of anxiety, or that [you] are not worth anything, or imposter syndrome.–Asian woman (20)

The receiver may not undergo as much harm as they should … may be due to constant exposure to microaggression, or if they don’t even know it’s a microaggression and that they should be offended.–Hispanic woman (26)

### 2. The meaning of microaggression can be affected by deliverers’ intent

Juxtaposing the relative importance of the harm experienced by receivers of microaggression, participants also found the deliverers’ intent to contribute to their perception of microaggressions. Specifically, participants believed that the presence of ill-intent to insult POC defined major discrimination whereas the absence of ill-intent defined microaggression. An 18-year-old African American man stated, “I think what makes it a microaggression is the lack of intention. I personally thought Vignette 4 is the definition of a microaggression because if someone is not *trying* to be racist, [but the exchange] still is [racist].” Similarly, a 22-year-old Asian man said, “Intent matters because if [deliverers] are trying to be open and confidently racist, it’s a lot worse than if they are doing it by accident. I know there’s been a lot of modern racism… because of societal norms.”

Whereas POC and White participants did not differ in their perspective regarding importance of intent in evaluating microaggressions, some participants indicated that microaggression should not be regarded as racism or taken seriously if the deliverers were not intentionally offending POC individuals. A 22-year-old Asian man commented on the nature of well-meaning comments and stated that he did not believe microaggression “should be taken as a slight.” Similarly, another participant considered microaggression to be a nonsensical concept. He said,

A world of political correctness will never happen in our lifetime. If there’s malicious intent—explicit or implicit—then it should just be categorized as racist and you should feel harm. It’d be just more so understanding that there are ignorant people out there in the world and you won’t be able to enforce your change on everyone’s minds.–Asian man (19)

Based on our observation, participants were in greater agreement that microaggressions reflected racial biases as long as the receivers considered them as such and were inappropriate. By contrast, participants were relatively more discrepant in their opinions about the importance of deliverers’ intent.

#### 2a. Microaggression reflects cultural ignorance

Data suggested that participants perceived microinsults and microinvalidations differently. They were more likely to consider microinsults (i.e., Vignettes 2 and 3) to be discriminatory and consider microinvalidations (i.e., Vignettes 4 and 5) to reflect deliverers’ cultural or racial ignorance. Similar to other themes and subthemes, we did not find systematic differences across POC and White participants’ responses; rather, the discussions reflected individual differences in attitudes toward discrimination. For example,

Microaggression is a clumsy interaction that foments an ignorant or toxic environment without knowing it.–Hispanic man (20)

I don’t think the receiver [in Vignette 4] was being rude… I feel like he is in a bubble that he’s trying to ignore the fact that racism does exist… he was being insensitive of where the Black student stood.–Black woman (21)

It comes from [a] lack of understanding of the cultural experience and the background of someone else.–Asian man (22)

#### 2b. Microaggression incidents offer opportunity for cultural learning

Extending beyond cultural ignorance, POC participants reported that they would take advantage of their microaggression experiences to educate the deliverers about the racialized reality of ethnic minority individuals. For example,

If they are being nonintentional, meaning they are just ignorant, then we should educate them on why they are being ignorant and possibly how to change their behavior. If they are being intentional, then there’s no room for change [and] no room for improvement… they could possibly go on just being microaggressive to other people—or even worse—just racist.–African American man (19)

When you grapple with the idea of racism, which includes discrimination and microaggression, you have to take in context everybody’s cultural background. Some people have grown up in communities where they have literally never seen anyone with a drop of melanin in their skin. That’s sad but that doesn’t mean that right off the bat we can shame them for just not knowing [and for] being ignorant. It places People of Color into a role where we may not necessarily want to be educators but it’s a good opportunity to take. I thought that may be [Vignette 4] wasn’t necessarily a direct microaggression but it’s a good place to start having a good conversation. I side with the Black student; you’re not Black you wouldn’t know. If this was a positive situation, the White student would say, ‘tell me.’–mixed heritage, Asian and White woman (20)

This subtheme emerged only from POC participants’ focus group sessions. Rather than ignoring or confronting the deliverers, responses suggested that POC participants would help others learn about the negative impact of racism. They hoped that these experiences could lead to productive conversations and prevent future perpetration of racism by the same deliverers. This view was not shared by all POC individuals, however.

#### 2c. Racism is a meta-construct that subsumes microaggression and major discrimination

Only a small group of POC and White participants considered microaggression to be categorically different from major discrimination. For example, a White man said, “I would say [discrimination and microaggression] are separate categories … because [the example in Vignette 1] is blatant.” By contrast, other POC and White participants tended to consider microaggression to be a less severe and subtler type of discrimination but not something that was completely distinct from racism. For example,

I don’t understand why we need all these new terms like racial microaggressions. It’s either racism or it’s not racism.–Asian man (22)

Other participants said,

I think [microaggression] is still discrimination, but kind of low-key and hard to see [at] the surface level.–African American woman (21)

Whether you can tell if it’s implicit or explicit really matters. If it’s explicit and [deliverers] are being outwardly racist, they are obviously being blunt about it. If it’s implicit… it’s still considered harmful or microaggression because it’s evidence of systemic discrimination.–Asian woman (19)

[Vignette 2] is an in-between of a microaggression and being racist because the statements were profoundly racist; I think most people would identify it as that but it’s not as direct as it was in the first scenario. It’s definitely not subtle enough to be a microaggression because anyone could have realized that those were racist statements.–White woman (20)

These responses suggested that participants thought of microaggression and major discrimination to reflect the same unified concept of racism, and that it would be inappropriate to discount the negative impact of microaggressions simply because these interracial exchanges were less severe than major discrimination events.

#### 2d. Collective experiences among People of Color and among Whites

Within the thematic category concerning deliverers’ intent, a subtheme emerged and highlighted the racialized experiences shared by POC and by Whites, respectively. The responses were shared equally by POC and White participants.

Sometimes White people say something and they offend or put people down because they never experienced that. They’ve never been in our shoes. They are not familiar with the feelings and psychological problem. If they were to know that, [then] they probably wouldn’t make us feel that way.–Hispanic woman (38)

I don’t know for sure if it’s intentional, but I think [deliverers] probably just don’t really want to believe about systematic racism… The fact that [the deliverer in Vignette 4] is a White American man—he has White privilege so it’s a lot easier for him to just say that because it doesn’t apply to him.–African American woman (19)

I think [microaggression] is just different racial experiences. In a predominantly White country, White people don’t really have to think about race too much.–Asian woman (18)

The culture of Whiteness in America is more or less the idea that everyone that’s White in America has this kind of built-in privilege that more or less has been propagated throughout the years… they do not have Black culture in the same way that Black people in America have a shared culture [and] a shared experience.–White man (25)

I think if you are the minority [then] you already feel like you’re at a disadvantage, so anything that anyone says might be taken more sensitively.–White woman (21)

These responses suggested that participants attributed microaggressions to White privilege and systemic racism, which differentially affected POC and White individuals. These collective racialized experiences facing different communities also could explain why White individuals might not consider how their unintentional actions could be offensive and insulting to POC individuals.

### 3. Context matters in how microaggressions are experienced and evaluated

A third prominent theme that emerged from our data concerned the importance of contexts that surround each racial microaggression incident. Participants suggested that their perspectives on each vignette might differ if they knew more about the deliverers’ intent. Relatively less frequently, participants also noted that how they evaluated microaggressions could depend on their understanding of the receivers’ perception and other nonverbal or situational cues. Some notable text illustrated this theme.

The deliverers’ intent is contingent on the context, like body language while you are determining what’s happening.–African American woman (21)

I honestly don’t want to speak for [receiver in Vignette 2] because I know how I would feel but there’s not enough context in this situation to say how he feels.–African American woman (21)

I thought [Vignette 3] was leaning toward a microaggression, although it was hard to tell for sure because you don’t know what the taxi driver was thinking… things like the other woman was just standing closer to the curb… At the same time, from the [receiver’s] point of view, it was the third time that had happened. Even if it wasn’t that specific taxi driver… it was probably [a microaggression in] at least one of the three incidents.–mixed heritage Asian and Hispanic woman (20)

I personally think that intent is largely inconsequential… context is king, and context is largely who the people are and what the result is.–White man (25)

The [deliverer in Vignette 2] was being racist but he was making jokes; the [receiver] was just rolling with it that’s what made it a microaggression. It was kind of brushed over by the [receiver]. I don’t know if [the deliverer] was well-intended or just making jokes; he might just have a very crass sense of humor.–African American man (19)

Both POC and White participants believed that contexts were important to what they thought about the vignettes: without knowing the receivers’ personal experiences, background information about the relationships between the deliverers and receivers, and environmental or historical factors made it challenging for everyone to have similar attitudes toward microaggressions. Although these beliefs were observed across participants of diverse backgrounds, the discussions were more prevalent among Whites than POC participants.

### 4. Receivers of microaggressions are considered “racist”

A fourth theme emerged from our data concerned the belief that POC receivers were “racist” and oversensitive to race should they find ambiguous interracial exchanges to be offensive or insulting. Although not shared by all participants, POC and White participants thought that the receivers—not the deliverers—in the microinvalidation vignettes were “racist” for calling out the occurrence of microaggressions and reacting negatively to these incidents. For example,

What I see is the Black student [in Vignette 4] basically bringing up race in an election and the White kid is basically saying that stuff doesn’t matter. To me, when I say ‘reverse microaggression,’ I basically mean that sometimes race doesn’t even come into a factor in people’s decisions but people look at it that way… none of that [race] was brought up.–White man (age unknown)

I think [the receiver in Vignette 5] is being racially microaggressive by blaming it all on White people and that’s how [Whites] get promoted. [She] is not really seeing White people as individuals [but rather] putting them into a whole group.–Hispanic woman (26)

### 5. Lived experiences shared by People of Color

Evident in focus group moderators’ field notes and from the interview responses, POC participants were more likely to report having had direct or vicarious microaggression experiences that were similar to the vignettes relative to White participants. Our analysis suggested that POC participants shared their lived experiences in order to illustrate their arguments. For example,

People always ask me where I’m from and when I say Dallas or Plano, they’re like ‘oh but where are you *really* from?’ I know the answer to that question and I know they are just trying to understand something, so I will correct them… I feel like you should correct people who say stuff like this or make assumptions about people based on race… I just take that as an opportunity to educate someone.–Asian woman (18)

If I got angry or let myself get upset every single time just based on how vitriol it gets I would absolutely have a mental breakdown.–African American man (18)

I said yes [to knowing the ill-intent of a deliverer] because I have been in this situation before—not with a police officer but a similar type of dialogue. In my experience, I was looking right at the person and I could see the intent in their eyes behind their smile.–African American man (21)

In response to Vignette 5 specifically, two participants said,

I related really hard to this vignette… that’s why I considered it more of a microaggression. I know that I have been passed over for promotion at a certain position because I am a female and pretty much in general I’m the only Person of Color in that particular workforce, even though I have got 5 to 8 years of experience on other people.–mixed heritage Asian and White woman (20)

I know my uncle changes his name on his resume so that [potential employers] won’t throw [his resume] out.–Asian woman (18)

We found that results from this theme validated the plausibility of our vignettes and the concept of racism—including both major discrimination and microaggression. The group differences in the rates by which POC and White participants shared their own racism-related experiences appeared to affirm the racial reality of POC individuals.

## Discussion

Using a mixed methods study design, this was the first psychological investigation of how POC and Whites evaluated the concept and examples of racial microaggression. The use of a focus group design facilitated in-depth discussions around the meaning of “microaggression,” defining features of this concept, and how individuals judge racial microaggressions [4, 50]. As the first study of this kind in psychology, participants’ qualitative responses indicated that individual and group differences in their attitudes toward racial microaggression reflected both personal and shared experiences with race and racism.

### Conceptualization of microaggression and its characteristics

The first three themes directly addressed our research questions regarding how people judged microaggression. In terms of participants’ conceptualization of microaggressions, participants tended to consider microaggression to be a form of racism, rather than a construct that was categorically distinctive from major discrimination. Participants thought that microaggressions occurred more frequently and exerted less severe negative consequences, as compared to major discrimination. Although POC have been shown to experience disproportionately higher rates of discrimination than their White peers [51], participants of diverse backgrounds were similar in the belief that racism and discrimination were undesirable. As seen in existing scholarly and public discourse [3, 52], our participants indicated that the word “micro-aggression” contributed to some confusion around the phenomenon. One participant (18-year-old Asian woman) suggested that microaggression might be an oxymoron because “micro” inadvertently trivialized interracial exchanges that were demeaning to POC groups and overlooked the underlying prejudicial messages. Participants also suggested that “aggression” implied a deliberate intent to harm, despite that many deliverers of microaggressions might be unaware of their racial biases and the potential negative impact of their actions on POC receivers.

### Decoding ambiguity: Roles of intent and harm

A key marker of microaggressions is the ambiguity in the deliverers’ intent and/or racist beliefs, and the psychological impact on the receivers [33]. Indeed, we observed individual differences in judgments about a range of racism- and microaggression-related events. Because it is often difficult to ascertain the deliverers’ intent and harm endured by the receivers, participants expressed that contexts were important in their forming of opinions about each microaggression vignette. Consistent with prior research, when interpersonal exchanges were ambiguous and when little information was available about the deliverers’ ill-intent, participants were prone to base their judgments on the psychological harm experienced by the receivers [35]. When a vignette showed explicit racial bias and differential treatment, there was a greater between-participant agreement that the exchange was discriminatory and therefore unacceptable. Relative to the major discrimination/microassault vignette, participants were less likely to consider the deliverer to be racist in the microinsult and microinvalidation vignettes. These differential attitudes toward major discrimination and microaggressions likely are attributed to greater uncertainty in terms of intent and harm surrounding microinsult and microinvalidation vignettes.

Our participants regarded the POC receiver’s experiences to be an important and credible source of information that shaped their judgments about microaggression vignettes. This finding appears to support the experiential validity of racial microaggressions [50]. Participants differed in how they interpreted the negative consequences facing the receivers, which highlighted the importance of considering for whom and under what conditions racial microaggression might be related to health outcomes [9]. Specifically, participants believed that individuals might vary in their reactions to the same microaggressive act for many reasons. According to the present focus group interviews, responses to ambiguous interracial exchanges, namely microinsults and microinvalidations, often draw on individual differences in attribution, stress reactivity, and prior experiences. This is because individuals appraise threats differently. People who are more uncomfortable with and intolerance of ambiguity may be more likely to recruit cognitive resources that are central to their self-concept and may recall prior experiences that are similar to the microaggressive acts [53]. For example, individuals who consider race to be more central to their personal identity were more likely to report racial discrimination and evaluate racism-related experiences negatively [54–56].

In our study, we did not define “harm” in specific ways. This was meant to elicit opinions from participants regarding their perception of harm. Participants considered harm to include not only immediate negative emotions (e.g., feeling annoyed), but also delayed psychological impact that could accumulate from repeated slights and denigrations. Although research indicated that microaggression experiences could lead to a range of deleterious psychological consequences including racial battle fatigue and cognitive burden [57–59], participants in our sample focused on negative emotions only. A small number of participants believed that microaggressions perpetuated structural and cultural biases against minoritized groups, and thus subtle racial discrimination was detrimental at the individual and societal levels.

### Strengths and empirical and practical implications

The use of a focus group design with supporting evidence from semantic differential ratings was a strength of this study. Although the use of a qualitative design and purposive recruitment of participants had been criticized for the possibly-conflating roles of demand characteristics and selection bias [3], we did not find evidence of these issues in this study. Specifically, approximately 10% of our participants had not heard of “microaggression” prior to this study. Even for those who have heard of the word, they did not all have a clear understanding of what microaggression means and participants varied in attitudes toward the concept.

There are several conceptual and practical implications associated with the present findings. Participants’ responses underscored the importance of contextualizing “microaggression” within the context of race and systemic racism. Consistent with challenges raised in previous research, our present participants find “microassault” to be similar to major discrimination [9, 27]. The meaning and lived experiences of microinsults and microinvalidations likely will be mischaracterized and trivialized outside of the broader nomological network of discrimination. Scholars have suggested replacing “microaggression” by other terms such as “inadvertent racial slights” [3]. According to some of the present participants, because of the confusions around “*micro*-*aggression*,” “racial insensitivity” or “cultural ignorance” may also be appropriate alternatives for characterizing microinvalidations experienced by POC. Contingent upon rigorous research clarifying the nomological structure of racism and discrimination-related experiences, “microaggression” and major discrimination may in fact be two lower-order elements of the same unified concept.

Results also highlight the importance of considering individual differences in the judgment of and responses to ambiguous interracial exchanges. Future research should continue to examine how individuals make sense of their experiences with racism and discrimination-related events, and how the deliverers’ intent may be perceived by a receiver or bystander. These psychological processes likely drive individual differences in responding to microaggressive acts and discriminatory events; subsequent studies can inform interventions that aim to train how receivers cope with these experiences or bystanders to engage in proactive antiracism [22, 60, 61].

### Limitations and future research directions

Despite the strengths and contributions, the present results should be considered with the following limitations in mind. First, the present research involved students at a predominantly White, private university in the Southwest, especially individuals who self-selected to participate in a study on racial microaggression. Our study was entitled “Understanding Racial Microaggression.” It was possible that the present participants were more curious and open to race talks than participants who did not sign up for a study such as this one. Students in higher education may be more aware, informed, and thoughtful of issues associated with microaggression than the general public. Prior research also showed that individuals who scored higher on need for cognition tended to be more analytical in evaluating discrimination-related vignettes [62]. Although an innovative start in this research endeavor, the present findings may be descriptive of the beliefs and experiences of university students at a predominantly White university in the Southwest region, and may not generalize to other individuals in higher education or the U.S. population. Future research should replicate these procedures to involve non-college, community adults to determine the degree to which our findings are generalizable across settings and segments of the general population.

Second, we did not measure participants’ own experiences with racial microaggressions nor their racial identity or ideology. Across focus groups, however, we observed varying levels of racial identity and colorblind beliefs across POC and White participants. Individual difference factors have been shown to shape people’s worldviews and reactions to interracial exchanges. For example, colorblind attitudes accounted for variability in how Whites evaluated racism and discrimination-related events; neuroticism also explained individual differences in the associations between discrimination and mental health outcomes [29, 63, 64]. Future research should systematically examine how individual difference factors including racial identity, colorblind attitudes, and prior discrimination-related experiences might shape people’s attitudes toward microaggressions and reactions to discrimination events.

Third, some of our written vignettes contained information about the receiver’s reactions whereas other vignettes did not. It is often unknown to the deliverer and/or bystanders regarding the degree to which POC receivers of microaggressions are negatively impacted immediately or at a later time. Relatedly, evident in our qualitative results, harm can be conceptualized in a number of ways including negative emotions, cognitive load, and internalized stigma. Future studies can standardize the vignettes presented to participants to account for systematic variations in the research stimuli and better examine the range of negative consequences of microaggressions.

## Conclusion

Participants tend to base their judgments of microaggressions on the receiver’s psychological experiences than the deliverer’s explicit prejudice or intent to harm. The present findings highlight the need for more research to consider individual differences such as personality, prior exposure to racism, and coping resources in the psychological responses to microaggression and other discriminatory acts. Echoing previous research, we argue that the concept of racial microaggression reflects undeniable experiential reality of many POC. The word “microaggression” may have caused confusion and debate that trivialize this form of racism. How microaggression relates to major discrimination and racism in a broader nomological network remains to be uncovered in future research.

## Supporting information

S1 Text Study procedures and focus group semi-structured interview script. (DOCX)

S2 Text Vignettes and semantic differential scales used in study materials. (DOCX)
