# Supplementary material for: Is microaggression an oxymoron? A mixed methods study on attitudes toward racial microaggressions among United States university students
Source: PLoS One. 2020 Dec 2;15(12):e0243058. doi: 10.1371/journal.pone.0243058 (PMC7710109; doi:10.1371/journal.pone.0243058)
Supplement: S1 Text — (DOCX) [file pone.0243058.s001.docx]

**Study Procedures and Focus Group Semi-Structured Interview Script**

Individual Tasks

- In this packet, you will see some scenarios describing interpersonal exchanges. Please read each scenario carefully and answer the questions following.
- When you are done, there are also several basic questionnaires. Please answer the questions carefully and honestly, but don’t spend too much time on any single question. I will collect the packet at the end of the study. You will keep the packet for the focus group discussion portion of the study.
- Please signal me when you have completed the questionnaires.

Focus Group Discussions

- Let’s begin by introducing your first name, your age, and your racial background. For example, you may identify as being Black, Hispanic, Asian, or White.
- This study is intended to study interracial interactions. By a show of hands, who have had experiences interacting with people of different racial backgrounds as your own?
- What comes to mind when we talk about racial discrimination? What are the defining characteristics of racial discrimination?
- What about racial microaggression? What comes to mind? What are the defining characteristics of racial microaggression?
- Each of you have just read are some scenarios that researchers have considered as racial microaggression.
  - By a show of hands, how many of you have heard of racial microaggression?
- Just so that we are on the same page, racial microaggression is defined by psychologists as subtle and sometimes conscious or unconscious verbal or nonverbal attacks to people of color. These incidents may communicate racial attacks, slights, or put-downs. Sometimes these incidents can be intentional, but sometimes can be well-meaning comments or gestures toward people of color such as Asian or Black Americans.
- [discuss each scenario] What make this scenario an example of racial microaggression? Do you think the deliverer [name in scenario] is intentional in being prejudicial to the receiver [name in scenario]? Do you think there is harm to the receiver in this incident?
- In these scenarios, the deliverers are all of White American backgrounds. Do you think the experience would differ if they are people of color?
- Overall, what do you think about the features associated with microaggression? To what extent do you think that deliverers’ intensions (whether they are explicit or implicit) matter, and that the receivers’ perceived harm matter? Please take about 2 minutes to consider them. You may write these down on a piece of paper. We will discuss as a group.
- Is there anything we should have discussed but didn’t?
