# Supplementary material for: Is microaggression an oxymoron? A mixed methods study on attitudes toward racial microaggressions among United States university students
Source: PLoS One. 2020 Dec 2;15(12):e0243058. doi: 10.1371/journal.pone.0243058 (PMC7710109; doi:10.1371/journal.pone.0243058)
Supplement: S2 Text — (DOCX) [file pone.0243058.s002.docx]

**Vignettes and semantic differential scales used in study materials**

Instruction: Below are several vignettes describing interpersonal encounters between two (groups of) people of different racial backgrounds, and the outcomes of these incidents. Please read the scenarios carefully, and then rate your opinions about each of them on a scale of 1 to 7.

1. A Mexican American heterosexual couple walked into a restaurant. The restaurant was relatively empty. Upon asking for a table, the waiter said to this couple, “We don’t serve people like you. We serve Americans.” The couple was offended and left to visit a different restaurant.
2. At a police precinct, a Black American man walked up to the police officer at the front desk. The White American police officer looked up, and said, “Are you here to turn yourself in for a crime?” The Black American, an accomplished attorney responded, “No, Officer Smith. I am attorney Brown. I am here to represent my client who got arrested this afternoon.” Officer Smith said, “Good for you! Did you become a lawyer after you have been arrested yourself?” Attorney Brown smiled and said, “Oh no! I never had been arrested or even suspected for a crime. I had always wanted to help promote justice that’s all.”
3. During rush hour in New York City, a White American woman and an Asian American woman, both dressed professionally, were waiting at the same spot waiting for a taxi. A White taxi driver saw them both and stopped to pick up the White woman. The Asian American woman thought to herself, “That’s the third time a taxi driver refused to pick me up. This is not cool!”
4. A racially diverse group of college students was discussing whether race had been a salient factor that influenced the most recent election in a Political Science class. A Black student said, “I think race played, and has always played, a critical factor in how people choose their candidates.” A White student said, “I disagree. Race is never a factor. When I think of race, I only think of the human race. I think only the candidates’ qualifications matter.” The Black student responded, “I don’t think you understand my racial experience.”
5. Two friends were talking about a recent promotion at their workplace. Julie, an Asian American woman who had been with the company said, “Only White people get promoted to leadership roles in this company. I don’t think that’s fair.” Julie’s friend, Tom, a White American man said, “I think everyone can succeed if they work hard enough. I think our company should only promote the most qualified people for the job.”

Semantic differential scales for each vignette:

a. **Microaggression**: This is an example of racial microaggression.

| 1 | 2 | 3 | 4 | 5 | 6 | 7 |
| --- | --- | --- | --- | --- | --- | --- |
| No |  |  | Undecided |  |  | Yes |

b. **Intent**: The deliverer:

| 1 | 2 | 3 | 4 | 5 | 6 | 7 |
| --- | --- | --- | --- | --- | --- | --- |
| Did not show differential treatment to the receiver(s) |  |  | Undecided |  |  | Differentially treated the receiver(s) |
| Did not deliver harm to the receiver(s) |  |  | Undecided |  |  | Delivered harm to the receiver(s) |
| Did not mean to insult or putdown the receiver(s) |  |  | Undecided |  |  | Was consciously intentional in insulting or putting down the receiver(s) |
| Was unaware of his/her treatment of the receiver(s) |  |  | Undecided |  |  | Was aware of his/her treatment of the receiver(s) |
| Was well-meaning in this incident |  |  | Undecided |  |  | Was intentionally hurtful in this incident |
| Was not racist |  |  | Undecided |  |  | Was racist |

c. **Harm**: The receiver(s):

| 1 | 2 | 3 | 4 | 5 | 6 | 7 |
| --- | --- | --- | --- | --- | --- | --- |
| Was not physically or psychologically harmed |  |  | Undecided |  |  | Was physically or psychologically harmed |

d. **Overall attitudes**: This encounter was:

| 1 | 2 | 3 | 4 | 5 | 6 | 7 |
| --- | --- | --- | --- | --- | --- | --- |
| Desirable |  |  | Undecided |  |  | Undesirable |
| Not stressful to the receiver(s) |  |  | Undecided |  |  | Stressful to the receiver(s) |
